# PACIC: patient activation and problem solving not related to age in patients with depressive symptoms

**DOI:** 10.1007/s00406-025-02061-5

**Published:** 2025-08-06

**Authors:** Karoline Lukaschek, H. Schillok, L. Junker, C. Jung-Sievers, P. Falkai, P. Henningsen, T. Dreischulte, G. Pitschel-Walz, H. Krcmar, A. Schneider, P. Schoenweger, C. Haas, V. Brisnik, F. Gökce, J. Eder, L. Pfeiffer, V. von Schrottenberg, C. Teusen, M. Bühner, J. Gensichen

**Affiliations:** 1 Institute of General Practice and Family Medicine, University Hospital, LMU Munich, Munich, Germany; 2 Graduate Program “POKAL—Predictors and Outcomes in Primary Care Depression Care” (DFG-GrK 2621), Munich, Germany; 3 Psychological Methods and Assessment, Department of Psychology, Ludwig Maximilian University of Munich, Munich, Germany; 4 Chair of Public Health and Health Services Research, Institute for Medical Information Processing, Biometry and Epidemiology, Pettenkofer School of Public Health, LMU Munich, Munich, Germany; 5 Pettenkofer School of Public Health, LMU Munich, Munich, Germany; 6 Department of Psychiatry and Psychotherapy, University Hospital, LMU Munich, Munich, Germany; 7 Department of Psychosomatic Medicine and Psychotherapy, Klinikum rechts der Isar, Technical University of Munich, Munich, Germany; 8 School of Computation, Information and Technology, Technical University of Munich, Garching, Munich, Germany; 9 Institute of General Practice and Health Services Research, School of Medicine, Technical University of Munich, Munich, Germany; 10 Institute of Psychology, University of the Bundeswehr Munich, Neubiberg, Germany

**Keywords:** Patient assessment of chronic illness care (PACIC), Chronic care model (CCM), Depression, General anxiety disorder

## Abstract

The Patient Assessment of Chronic Illness Care (PACIC) assesses alignment of chronic care with the Chronic Care Model (CCM). We analysed PACIC subscales in patients with depressive symptoms to identify gaps in patient-centred care. A total of *N* = 2741 patients (59.5% women, mean age 45.3 ± 16.9) were assessed for depression (PHQ-9), anxiety (GAD-7) and socioeconomic data. PACIC data from 1210 patients (62.7% women, mean age 47.2 years ± 16.8) revealed low scores in subscales patient activation, goal setting, problem-solving, and follow-up. Patient activation and problem-solving were age-independent. Overall, the low PACIC scores highlight poor CCM alignment in German depression care.

## Introduction

The Chronic Care Model (CCM) is an evidence-based framework that supports collaborative management of chronic diseases such as depression [1]. It promotes coordinated, patient-centred care through a productive partnership between an informed patient and a proactive healthcare team [2]. This interaction is driven by four key elements: self-care, including self-management and behavioural activation; coordination through teamwork and case management; decision-making support based on evidence-based guidelines; and the use of IT and data from clinical and practice records [3]. The Patient Assessment of Chronic Illness Care (PACIC) assesses the alignment of chronic care with principles of the CCM from the patient’s perspective [4–7]. Higher PACIC scores indicate better perceived alignment of care with the CCM. Different PACIC versions vary in items and response formats [8], but all cover five subscales: (1) patient activation, (2) delivery system and decision support, (3) goal setting, (4) problem-solving and contextual counselling, (5) follow-up and coordination.

The research training group POKAL (PrädiktOren und Klinische Ergebnisse bei depressiven ErkrAnkungen in der hausärztLichen Versorgung) aimed at improving the care of multimorbid patients with depressive disorders in general practice and at qualifying next generation scientists to work in this field by employing the CCM as a working model and developing it further.

In order to enable research questions beyond those targeted by individual POKAL projects to be investigated– which are e.g. somatic disorder and personality disfunction [9, 10], validation of new questionnaires on depression [11] and suicidality [12, 13], effective components of collaborative care [14], and deprescribing of antibiotics [15]—the POKAL consortium has systematically developed and collected a core data set from all research participants. It contains basic socio-demographic, previous history of mental health, and current medication [3] and [16] The present cross-sectional analysis of the POKAL core data set (POKAL-CDS) examines patients’ assessments of chronic illness care in depression treatment across inpatient and outpatient settings [3]. Instead of an overall PACIC score, we focus on its subcategories [17] to highlight strengths and weaknesses in specific care aspects and patient-provider collaboration. The findings may inform targeted improvements, including potential age- or sex-specific adaptations. Care needs and preferences vary across age and sex [18]. Older adults may prioritise coordination, while younger patients may prefer digital and flexible care [19, 20]. Women often report higher symptom burden and seek more relational support, whereas men may benefit from structured, goal-oriented approaches. Tailoring care can thus improve engagement and CCM alignment.

## Methods

### Setting and sample description

A total of *N* = 2741 patients aged ≥ 15 years, proficient in German, who participated in one of five POKAL sub-studies and completed the POKAL-CDS [3, 21], were recruited from outpatient (*n* = 2438; 89%) or inpatient (*n* = 303; 11%) settings and through social media between 01/2022 and 07/2024 in Southern Germany and Austria. The five sub-studies are described in greater detail elsewhere [16]. In brief, they include: a study on identifying predictors of depression severity; two studies validating new questionnaires on depression and suicidality, respectively; a study on psychoeducation in primary care; and a study exploring the association between somatic symptom disorder and personality dysfunction. Most participants were female (*n* = 1640; 59.5%), mean age was 45.3 years (± 16.9 years; range: 15–94 years).

As each of the 5 POKAL sub-studies involved human participants, they were approved by the respective Medical Ethics Committees of the Medical Faculty, LMU Munich and Technical University Munich/University Hospital Klinikum rechts der Isar.

### Measures

Participants reporting a chronic illness (lasting ≥ 3 months and affecting them physically, emotionally, or socially) completed the PACIC questionnaire, which assesses their experience of chronic illness care over the past 6 months. We used the 11-item PACIC version [22, 23] with four response categories 0–25%; 26–50%; 51–75%; 76–100% (0%=“never”, reflecting low perceived care, and 100% “always”, indicating high perception of care aligned with chronic illness management principles).

The Patient Health Questionnaire-9 (PHQ-9) assesses nine key criteria used for diagnosing depressive disorders [24]. Patients rate symptoms over the past two weeks on a 4-point Likert scale (0 = “Not at all” to 3 = “Nearly every day”), yielding a total score of 0–27. Higher scores indicate greater depression severity. Categories include no depression (0–4), mild (5–9), moderate (10–14), moderately severe (15–19), and severe (20–27). A score of ≥ 10 suggests possible major depressive disorder [25]. 

The Generalized Anxiety Disorder 7-item scale (GAD-7) assesses anxiety severity over the past two weeks [26]. Participants rate each item on a 4-point Likert-type scale (0 = “Not at all” to 3 = “Nearly every day”), yielding a total score of 0–21. Higher scores indicate greater anxiety. Categories include minimal (0–4), mild (5–9), moderate (10–14), and severe (15–21) anxiety. A score of ≥ 10 suggests possible generalized anxiety disorder [27]. 

Additionally, socio-economic data (age, sex, marital status, level of education, employment status, place of residence) were collected.

### Data processing

Participants with no PACIC data at all were removed from further analyses. Thus, our data set consisted of 1035 complete and 175 partially complete cases regarding PACIC. To make use of all data, for 175 partially available PACIC scores, missing PACIC items were imputed with MICE [28]. Covariates used for imputation were age, sex, employment status and other psychometric measures (GAD, PHQ). For the descriptive analyses, the mean values of the PACIC subgroups were calculated. For each PACIC subgroup, a generalized additive model (GAM) was modelled using a p-spline basis [29]. P-splines are a non-linear way to model a continuous relationship between two numeric covariates under the assumption that a smoother function is more probable than a wiggly one [30]. 

## Results

A total of 1210 patients (mean age: 47.2 years, ± 16.8; Range: 16–89 years, women: *n* = 753, 62.7%) with PACIC information were included in the present analysis. Characteristics of the total PACIC sample are shown in Table 1. A total of 247 patients had a moderate depression severity (PHQ-9 ≥ 10) and a total of 438 patients had a moderate anxiety severity (GA-7 ≥ 10). Women were slightly younger than men (46.9 years ± 16.7 compared to 48.0 ± 16.4), and had higher PHQ-9 (mean women: 5.34 ± 5.72; mean men: 5.24 ± 6.02) or GAD-7 (mean women: 8.19 ± 5.54; mean men: 7.39 ± 5.75) scores. A moderate depression severity (PHQ-9 ≥ 10) was found in 21.4% (*n* = 86) of men and 21.0% (*n* = 150) of women. A moderate anxiety severity (GAD-7 ≥ 10) was found in 32.7% (*n* = 133) of men and 38.6% (*n* = 279) of women. Men were more often in a partnership (60.0% compared to 55.8% of the women) or currently employed (61.6% compared to 55.6% of the women).

**Table 1:** PACIC sample description (*N* = 1210)

	*n* (%)	Mean/SD
Sex (missing = 28) Female Male Divers	1182 753 (63.7) 415 (35.1) 14 (1.2)	
Age (missing = 40)	1170	47.15 ± 16.78
PHQ-9 (missing = 70)	1140	5.36 ± 5.89
GAD-7 (missing = 41)	1169	8.0 ± 5.56
Children (missing = 87) no yes	1123 540 (48.0) 583 (52.0)	
Relationship status (missing = 62) Partnership single/divorced/widowed	1148 654 (57.0) 494 (43.0)	
Current Employment (missing = 22) no yes	1188 471 (39.7) 717 (60.3)	

### PACIC subcategories

**Fig. 1:**
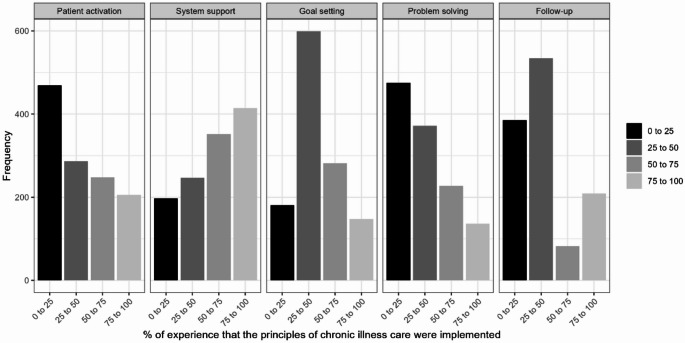
Distribution of Responses Across PACIC subgroups. 0% indicating no experience of chronic illness care principles being implemented, and 100% indicating constant experience of these principles being implemented

**Fig. 2:**
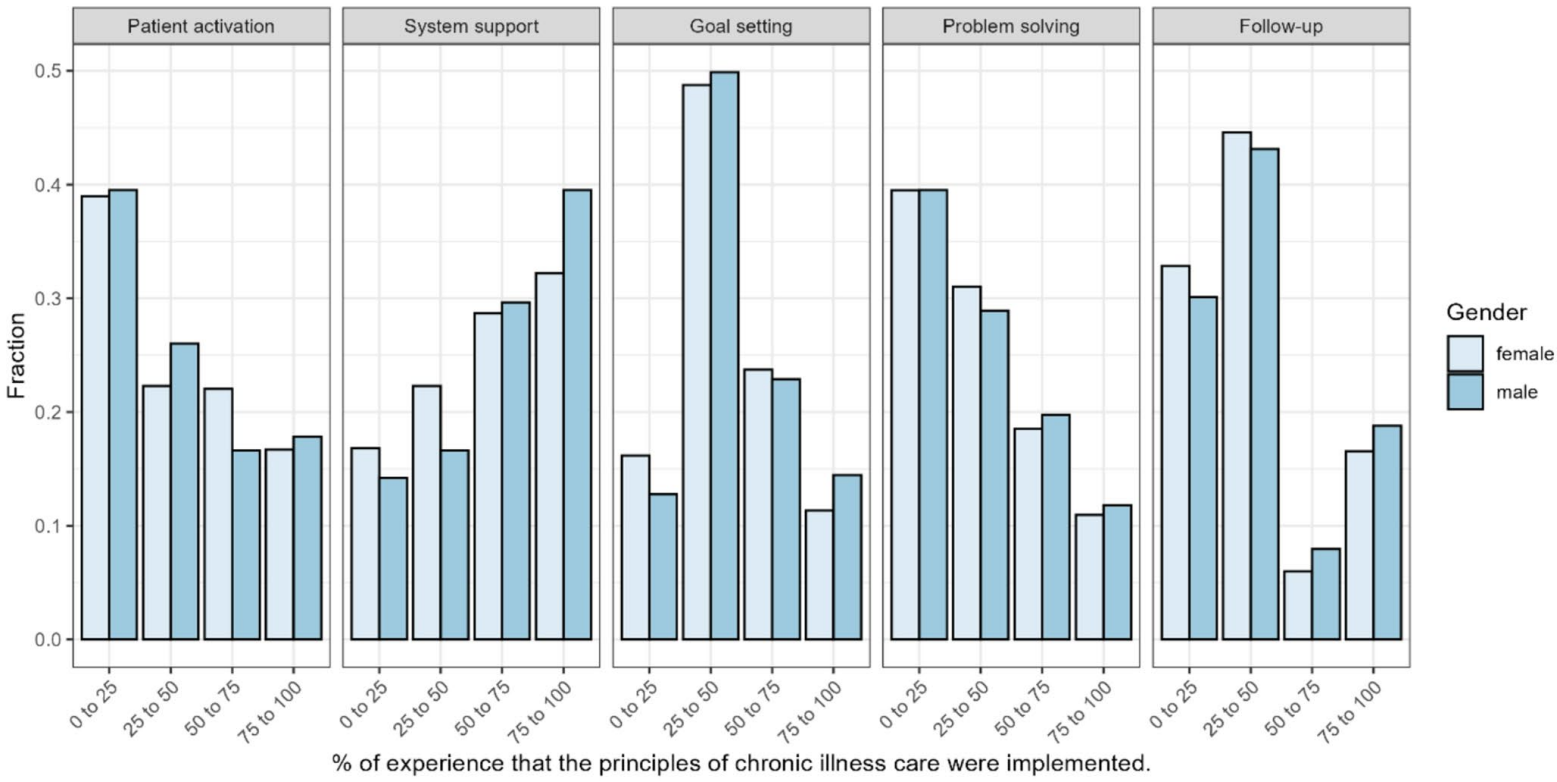
Distribution of Responses Across PACIC subgroups by sex. 0% indicating no experience of chronic illness care principles being implemented, and 100% indicating constant experience of these principles being implemented

Patients reported low experience in the PACIC-domains of patient activation, goal setting, problem solving, and follow-up (Fig. 1). However, 34.2% (*n* = 414) reported high experience with delivery system and decision support. Figure 2 shows the PACIC-domains by sex. Although both sexes show a rather similar pattern, a notably higher proportion of male participants reported experiences within the 75–100% category in the PACIC subscale “system and decision support”.

**Fig. 3:**
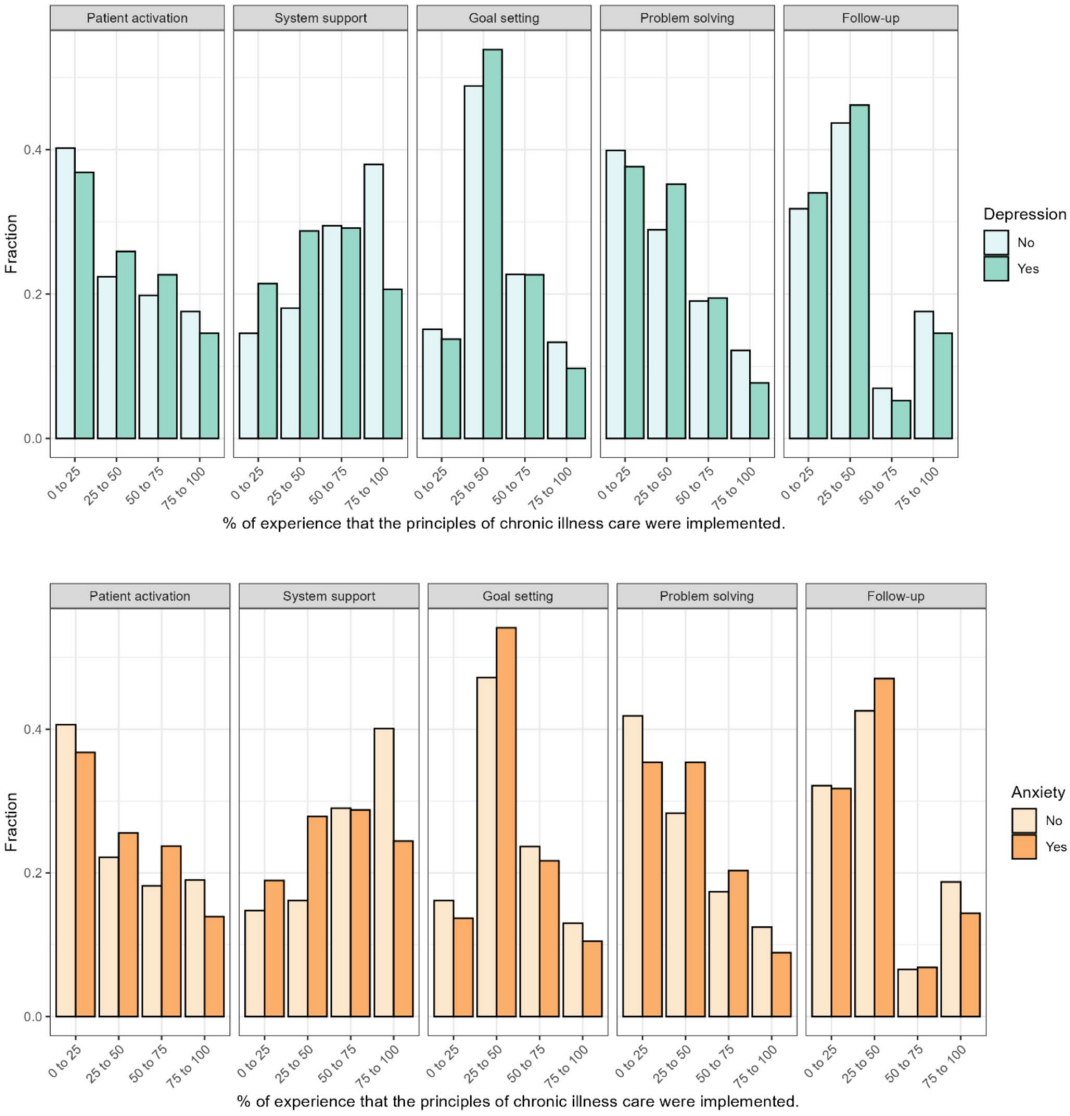
Distribution of Responses Across PACIC subgroups by depression (upper part) and anxiety (lower part). 0% indicating no experience of chronic illness care principles being implemented, and 100% indicating constant experience of these principles being implemented

As shown in Fig. 3, there were no major differences between participants with depression or anxiety compared to participants without depression or anxiety. However, the former had a slightly higher experience with goal setting and a slightly lower experience with delivery system and decision support.

**Fig. 4:**
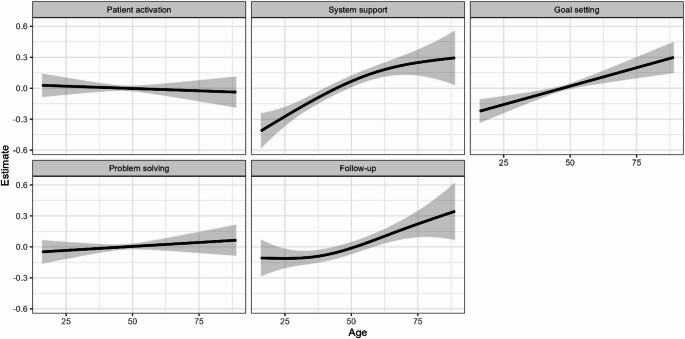
P − Spline Model with random intercept for study. Note: There was a considerable variance in patient response across all ages and groups that is not plotted due to visibility

Figure 4 shows how PACIC subgroups were related to age. The smooths and their respective confidence intervals indicate that System Support, Goal Setting and Follow-Up increase linearly with age. Patient activation and Problem Solving appear not to be related to age.

## Discussion

In our sample the patient assessment of chronic illness care was rather low in all subdomains of the PACIC. This indicates that patients perceive their care as poorly aligned with the CCM. Specifically, it suggests deficiencies in key areas such as patient activation, goal setting, problem-solving, follow-up, and overall coordination of care. This can imply limited patient engagement, insufficient support for self-management, or inadequate integration of chronic care elements into their treatment plan [31]. Low PACIC scores may highlight unmet needs in delivering person-centred, coordinated care and reflect gaps in shared decision-making, tailored counselling, and collaborative goal-setting between patients and healthcare providers.

Both sexes showed similarly low PACIC scores across subdomains, indicating poor CCM alignment overall. Women reported a slightly lower experience than men in the domain of delivery system design and decision support, while no substantial gender differences were observed in other areas. PACIC-subdomains “patient activation” and “problem solving” were not associated with age. The association between age and PACIC has been investigated in various studies, showing no or only slight correlations [4, 32]. Some studies report that age shows weak to moderate correlations with specific subscales of the PACIC [33]. For example, older patients tend to score higher on subscales like follow-up/coordination and problem-solving/contextual counselling. This may be attributed to their frequent interactions with healthcare providers due to chronic illness or longer duration of illness. Thus, it has been suggested that age-specific strategies may enhance patient experiences, especially in areas like coordination and problem-solving support. However, the association between problem-solving ability and age is rather complex [34], influenced by different types of cognitive skills and contexts: Regarding everyday problem-solving, middle-aged adults often outperform younger and older adults by demonstrating greater effectiveness in practical, context-specific scenarios, likely due to life experience and accumulated knowledge [35]. They prioritise emotional well-being, adopting strategies aligned with socioemotional goals [36]. 

Age-specific approaches to enhance quality of care may include: For younger adults, digital tools and peer-based formats may support engagement. Middle-aged adults might benefit from flexible, wortk-life compatible interventions and pragmatic problem-solving support. For older adults, strengthening care continuity and coordination appears particularly relevant, along with simplified self-management tools adapted to potential cognitive or sensory limitations. Tailoring care processes to age-specific needs could help improve alignment with CCM principles across the lifespan.

### Strengths and limitations

Our major strength is the POKAL research network, which provided access to 1,210 patients. As limitations, we performed descriptive analyses only. The scaling of our PACIC answer categories does not allow for fine-grained analyses. Our findings are based on a large sample from Southern Germany and Austria, which may limit generalisability to other regions. The overrepresentation of women could reflect selection bias related to help-seeking behaviour. Moreover, due to recruitment strategies, the general population is not fully reflected.

## Conclusion

PACIC scores were low across all subdomains, indicating poor alignment with the CCM and gaps in patient engagement, decision-making, and care coordination. “Patient activation” and “problem-solving” showed no association with age. While older patients may score higher in follow-up and coordination due to frequent healthcare interactions, problem-solving abilities vary with age, shaped by cognitive changes, experience, and emotional priorities.
